# Effect of Different Sugar Beet Pulp Pretreatments on Biogas Production Efficiency

**DOI:** 10.1007/s12010-016-2279-1

**Published:** 2016-10-20

**Authors:** Krzysztof Ziemiński, Monika Kowalska-Wentel

**Affiliations:** Faculty of Biotechnology and Food Sciences, Institute of Fermentation Technology and Microbiology, Lodz University of Technology, 171/173 Wolczanska Str, 90-9254 Lodz, Poland

**Keywords:** Anaerobic digestion, Grinding, Thermal-pressure pretreatment, Enzymatic hydrolysis, Gompertz model

## Abstract

The objective of this study was to determine the effect of different sugar beet pulp (SBP) pretreatments on biogas yield from anaerobic digestion. SBP was subjected to grinding, thermal-pressure processing, enzymatic hydrolysis, or combination of these pretreatments. It was observed that grinding of SBP to 2.5-mm particles resulted in the cumulative biogas productivity of 617.2 mL/g volatile solids (VS), which was 20.2 % higher compared to the biogas yield from the not pretreated SBP, and comparable to that from not ground, enzymatically hydrolyzed SBP. The highest cumulative biogas productivity, 898.7 mL/g VS, was obtained from the ground, thermal-pressure pretreated and enzymatically hydrolyzed SBP. The latter pretreatment variant enabled to achieve the highest glucose concentration (24.765 mg/mL) in the enzymatic hydrolysates. The analysis of energy balance showed that the increase in the number of SBP pretreatment operations significantly reduced the gain of electric energy.

## Introduction

An increase in production of electric energy from renewable resources has been one of principal objectives in European Union countries. In line with global trend, the energy supply from biogas has been dynamically growing in Poland over the last years. Production of biogas by agricultural plants increased by 137.29 million m^3^ between 2011 and 2014 [1]. In 2015, 358,015 × 10^6^ m^3^ biogas was produced in Poland by 85 agricultural plants with the overall installed capacity of 134 MW [1]. Further development of these plants depends on the availability and price of substrates for anaerobic digestion. Inexpensive and abundant agro-industrial wastes and by-products are particularly attractive streams for biogas generation and their utilization by means of conversion to biogas is environmentally-friendly.

One of attractive and inexpensive substrates for biogas production is sugar beet pulp (SBP), which is a residue remaining after sucrose extraction. Conversion of 1 t of sugar beet roots generates around 70 kg SBP dry weight [2]. Only in EU countries, around 13.34 × 10^6^ t of sucrose were produced from sugar beets harvested in 2014 that caused accumulation of nearly 4.43 × 10^6^ t of SBP dry weight [3]. In 2012, a biogas plant with the installed capacity of 2 MW, producing biogas from fresh and ensiled SBP (50 × 10^3^ t a year), was located in the sugar factory in Strzelin (Poland). Investments in plants using SBP for biogas generation are thought to increase.

Biogas yield from anaerobic digestion depends on the construction of digesters, technological parameters and chemical composition of substrates. Degradation of polysaccharides contained in lignocellulosic biomass to simple sugars is the rate-limiting step of this process. SBP contains around 22–30 % cellulose, 22–30 % hemicelluloses (primarily arabinans), 24–32 % pectin, and 1–3 % lignin on a dry weight [4]. The presence of lignin and crystalline cellulose as well as the limited access of hydrolytic enzymes to cellulose and hemicellulose chains are the main bottlenecks responsible for the slow degradation of lignocellulosic biomass under anaerobic conditions and low biogas yields [5]. Therefore, the development of efficient depolymerization methods is necessary to increase biofuel production from lignocellulosic materials.

Promising techniques of plant biomass degradation are thermohydrolysis and enzymatic hydrolysis. The latter outperforms chemical depolymerization because of the high substrate specificity of enzymes and mild process conditions. Furthermore, it may be applied in industrial scale and enables savings of chemicals and energy [6]. The cost-effectiveness of enzymatic hydrolysis may be increased by application of more active enzyme preparations and favorable changes in the structure and crystallinity of lignocellulose, making it more susceptible to the action of enzymes. Therefore, mechanical or thermal pretreatments before enzymatic hydrolysis, which affect the crystallinity of cellulose, play an important role. Grinding of lignocellulosic materials affects their structure and reduces the crystallinity of cellulose, making it more susceptible to enzymatic depolymerization [7]. It was found that this method may increase glucose yields from enzymatic hydrolysis by even 60 % [8]. Also, pretreatment at elevated temperature has been increasingly applied for partial degradation of plant biomass. However, thermal pretreatment above 160 °C may cause partial conversion of polysaccharides and lignin to phenolic or heterocyclic compounds like vanillin, vanillic alcohol, furfural, and hydroxymethylfurfural (5-HMF) [9]. These substances may inhibit the growth of microorganisms mediating anaerobic digestion [10]. To reduce their formation, thermal biomass pretreatment needs to be conducted under mild conditions. In the latter case, it is usually combined with mechanical and/or enzymatic pretreatments, not only to enhance biogas yield but also make its production cost-effective. Biomass pretreatment conditions have to be optimized because its costs are one of important factors deciding of the economic gain from biogas production.

This study aimed at comparison of effects of different SBP pretreatments (grinding, thermal-pressure and enzymatic as well as their combinations) on biogas yield from anaerobic digestion and evaluation of the economic feasibility of these processes.

## Material and Methods

### Sugar Beet Pulp and Inoculum

In this study, biogas was produced from fresh SBP, which was obtained from the sugar factory owned by Südzucker in Strzelin (Poland), using an anaerobic sludge as an inoculum. The sludge was harvested from an agricultural biogas plant, which was supplied with SBP and concentrated by sedimentation in an Imhoff funnel for 24 h to increase the concentration of microbial cells in the inoculum. Then, the sludge was kept for 4 weeks at 37 °C until methane evolution was ceased. Parameters of SBP and inoculum (Table 1) were determined as described in the section “Analytical Methods.”

**Table 1:** Parameters of sugar beet pulp and inoculum

Biomass composition		Sugar beet pulp	Anaerobic sludge (inoculum)
TS	%	25.63 ± 4.00	9.94 ± 1.40
VS	% TS	94.50 ± 2.25	72.94 ± 1.20
Ash	% TS	1.40 ± 2.25	27.06 ± 1.20
COD	gO_2_/kg TS	1107.09 ± 138.37	994.5 ± 64.00
TOC	g/kg TS	393.40 ± 8.05	395.79 ± 9.00
VFA	g CH_3_COOH/kg TS	6.26 ± 0.46	4.28 ± 0.30
TKN	g/kg TS	97.20 ± 1.80	40.42 ± 1.46
Phosphorus	g/kg TS	3.13 ± 0.70	10.91 ± 2.86
pH	–	5.43 ± 0.01	6.05 ± 0.01
Cellulose	% TS	29.50 ± 1.40	n.d.
Hemicellulose	% TS	27.51 ± 0.60	n.d.
Pectin	% TS	22.80 ± 1.42	n.d.
Lignin	% TS	3.82 ± 0.70	n.d.

*n.d.* not determined

### Enzyme Preparations

Enzymatic hydrolysis of SBP was conducted using a mixture of commercial preparations: Celustar XL and Agropect pomace (3:1), which are described in Table 2.

**Table 2:** Activities of enzyme preparations

Preparation	Enzyme source	Main activities
Celustar XL ^a^	*Trichoderma longibrachiatum*	endoglucanase, xylanase activity of 15,000–17,000 U/g
Agropect pomace^b^		pectinase

### Mechanical Pretreatment

SBP was ground using a Sprout Waldron 374 disk mill, with a 3-kW engine. An average size of the ground SBP particles, which was determined using sieves, was around 2.5 mm.

### Thermal-Pressure Pretreatment

Samples of ground and not ground SBP were suspended in tap water (the ultimate solid substance concentration was around 10 % *w*/*v*) and thermal-pressure pretreated at 120 °C under the pressure of 4 bars for 10, 15, and 20 min, using an ASL 80 M autoclave.

### Enzymatic Hydrolysis

To determine the impact of grinding and duration of thermal-pressure pretreatment on the susceptibility of SBP to enzymatic degradation, samples of not pretreated (EP), ground (MEP), thermal-pressure pretreated (TEP), or ground and thermal-pressure pretreated (MTEP) pulp, which were suspended in tap water and contained 10 % *w*/*v* dry weight, were subjected to enzymatic hydrolysis at 50 °C for up to 4 days, using the mixture of Celustar XL and Agropect pomace (3:1). The dose of the enzymes (0.15 IU/g total solids (TS)) was optimized in a previous study [11]. The process of enzymatic hydrolysis was monitored by quantification of reducing sugars released from the polysaccharides contained in SBP.

### Experimental Setup

Batch anaerobic digestion processes were conducted under mesophilic conditions (37 °C) with stirring (4 rpm) in two identical 1-L (the working volume) glass digesters. Before each process, these digesters were flushed for 3 min with a mixture of nitrogen (75 % *v*/*v*) and carbon dioxide (25 % *v*/*v*) to ensure anaerobic conditions. Volumes of biogas synthesized daily were measured using a system consisting of an electronic Aalborg® GFM17 flow meter, governed by a computer system. The same system was used for the continuous temperature measurements during anaerobic digestion. The constant temperature was maintained using a thermostat connected to a water jacket of each digester. Before anaerobic digestion, the pH of each batch of SBP was adjusted to around 7.2 using Na_2_CO_3_. To initiate the digestion process, the inoculum derived from the agricultural biogas plant was added to the substrates in a dose of 20 g TS/L.

Each process of anaerobic digestion was conducted in duplicate, and the results are presented as means ± standard deviation.

Parameters of anaerobic digestion of pulp samples (not pretreated (WP), –ground (MP), enzymatically hydrolyzed for 2 days (EP), ground and enzymatically hydrolyzed for 2 days (MEP), thermal-pressure (for 15 min) and enzymatically (for 2 days) pretreated (TEP), and ground, thermal-pressure (for 15 min) and enzymatically (for 2 days) pretreated (MTEP)) are presented in Table 4.

### Kinetic Model of Biogas Production

Kinetics of biogas production was modeled using a modified Gompertz equation [12]. It was assumed that in batch processes, biogas production was affected by the specific growth rate of microorganisms in a digester [13]. The modified Gompertz equation is as follows:

1 $$ y(t)=A.\mathit{\exp}\left\{- \exp \left[\frac{\mu e}{A}\left(\lambda -t\right)+1\right]\right\} $$

where *y* is the biogas accumulation (mL/g volatile solids (VS)) at time *t* (day) and *t* is the time (day) over the digestion period. *A* is the biogas production potential (mL/g VS), *μ* is the maximal biogas production rate (mL/g VS day) while *λ* is the lag phase (day) or minimum time between the inoculation and biogas appearance, and *e* is a mathematical constant (2.718282). Kinetic constants *A*, *μ*, and *λ* were determined using the nonlinear regression and Matlab software [14].

### The Efficiency of Converting Biogas to Energy

Factors deciding of pretreatment conditions of lignocellulosic materials used to produce biogas are as follows: the sort of biomass, costs of installation, and the potential gain of electric energy obtained from biogas [15]. In this study, the analysis of energy balance was based on comparison of energy which has to be used for pretreatment of SBP and energy produced by incineration of biogas derived from anaerobic digestion. It was assumed that biogas would be incinerated using the combined heat and power (CHP) system of 40 % electric efficiency and 43 % heat efficiency [16]. This analysis was based on outcomes of SBP pretreatments and biogas production in laboratory conditions. The electric energy gain from biomass was calculated from the equation:

2 $$ E=EVM\times n\times {V}_{CH4}\times M $$

where *E* is the energy value of biomass (kWh), EVM is the energy value of methane 10 kWh/m^3^, *V*_CH4_ is an increase in methane biosynthesis yield after pretreatment (m^3^/Mg), *M* is the weight of biomass (daily, monthly, annually) (Mg), *n* is the electric efficiency of cogeneration engine (electric energy) 40 %, and *n* is the heat efficiency of cogeneration engine (heat energy) 43 %.

The electric energy requirements for grinding of SBP were calculated based on the power of the electric mill and time of grinding:

3 $$ {E}_m=P/W $$

where *E*_*m*_ is the electric energy requirements of the mill (kWh/Mg), *P* is the power consumption by the mill (kW), and *W* is the grinding capacity of the mill (Mg/h).

The heat requirements for enzymatic hydrolysis (the temperature of batches of SBP was increased from 25 to 50 °C, and then, the temperature of 50 °C was maintained for 2 days) and thermal-pressure pretreatment (at 120 °C for 15 min) were calculated from the following equation [17]:

4 $$ {H}_s=\left\{\Big[\ \left(1/{q}_s\times {C}_p\times \left({T}_{\mathrm{final}}\hbox{--} {T}_{\mathrm{initial}}\right)\right]/3600\right\}\times 1000 $$

where *H*_*s*_ (kW h/t TS) is the heat required for the thermal pretreatment of the substrate; *q*_*s*_ is the content of solids in the substrate suspension (kg TS/m^3^); *C*_*p*_ is the specific heat capacity of the substrate, assumed equal to the specific heat capacity of water (4.18 kJ/kg/C); *T*_initial_ is the initial temperature of the substrate suspension, assumed as 25 °C; *T*_final_ is the final temperature of the substrate suspension, assumed as 50 °C or 120 °C, 3600 is the conversion factor between kJ and kWh.

### Analytical Methods

TS, VS, ash, volatile fatty acids (VFA), total organic carbon (TOC), total Kjeldahl nitrogen (TKN), and phosphorus were quantified by standard methods [18]. Reducing sugar concentration was determined using the alkaline 3′,5′-dinitrosalicylic acid (DNS) solution [19]. Cellulose was quantified by the acid-base method based on dissolving of lignin and hemicelluloses in nitric acid and sodium hydroxide, and gravimetric determination of cellulose dry weight [20]. Hemicelluloses were quantified by Ermakov method [21] based on their hydrolysis to monosaccharides that were assayed using the alkaline DNS reagent, according to Miller [19]. Pectins were assayed as calcium pectate according to [22]. Lignin was quantified according to the NREL standard laboratory analytical procedure (LAP) for determination of structural polymers in biomass [23]. Soluble sugars contained in enzymatic hydrolysates were quantified by HPLC using a Dionex ICS-3000 chromatograph equipped with a HPAEC detector and a Carbo Pac PAI column (4 × 250 mm). The isocratic elution of sugars was carried out using 16 mM NaOH (at 30 °C and at a flow rate of 1.0 mL/min). The volume of injected samples was 25 μL. Methane and carbon dioxide concentrations in biogas were determined by GC, using an Agilent 7890A GC chromatograph equipped with a TCD detector and a 2D column system (connected by a pneumatic switch): molecular sieve 5 A, 60/80 mesh 6 ft. × 1/8 in and Porapak Q 80/100 mesh, 6 ft. × 1/8 in.

## Data Analysis

Data analysis was performed using Statistica 12. The significance levels of different treatments between the samples were determined by one-way analysis of variance (ANOVA).

## Results and Discussion

### Substrate Characteristics

Chemical composition of fresh SBP, which was used in this study, is presented in Table 1. SBP contained 74.37 % moisture while organic substances accounted for 94.50 % dry weight. This composition is consistent with data reported by other authors [24, 25]. Cellulose and hemicelluloses constituted 29.50 and 27.51 % TS, respectively. The high content of structural polysaccharides that are slowly degraded under anaerobic conditions may negatively affect biogas yields from anaerobic digestion [6]. To enhance this process, plant biomass is pretreated before digestion [26]. Another parameter deciding of the susceptibility of vegetal biomass to biodegradation is the content of lignin [27], which in SBP is much lower (3.82 % TS) in comparison to many other lignocellulosic materials.

### Mechanical Pretreatment and Enzymatic Hydrolysis

**Fig. 1:**
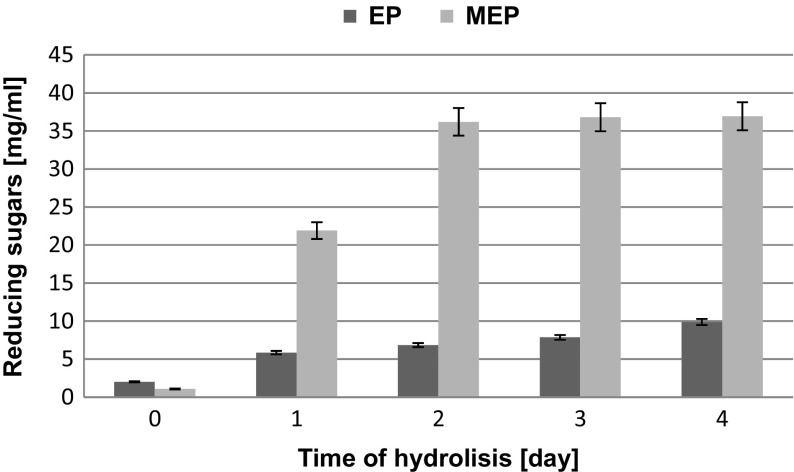
Changes in reducing sugars concentration during enzymatic hydrolysis of ground and not ground SBP

To determine the effect of SBP grinding on the concentration of reducing sugars in enzymatic hydrolysates, batches of the ground (dimensions of around 2.5 mm) and not ground (the control) SBP were treated with the mixture of hydrolytic enzymes under the same conditions. The size of ground SBP particles was selected based on results of preliminary experiments, which showed that it was optimal (unpublished data). The results of enzymatic hydrolysis are presented in Fig. 1 and Table 3.

**Table 3:** Concentrations of monosaccharides released within 2 days of enzymatic hydrolysis of sugar beet pulp

	Arabinose	Xylose	Glucose	Galactose	Mannose
	(mg/mL)				
EP	0.895 D (0.074)	1.998 C (0.105)	3.000 D (0.011)	0.006 C (0.002)	0.923 D (0.018)
TEP	11.211 B (0.121)	9.546 B (0.189)	23.116 B (0.143)	3.670 B (0.118)	5.003 B (0.233)
MEP	6.582 C (0.522)	9.284 B (0.206)	16.069 C (0.144)	0.220 C (0.009)	3.030 C (0.148)
MTEP	12.951 A (0.286)	10.830 A (0.231)	24.765 A (0.117)	4.340 A (0.197)	5.982 A (0.150)

Different letters (A, B, C, D) within the same parameters indicate statistical differences (*p* < 0.05). The values in brackets mean standard deviation

*EP* SBP not ground, *MEP* SBP ground, *TEP* SBP not ground after thermal pretreatment, *MTEP* SBP ground after thermal pretreatment

Grinding increases the specific surface of lignocellulosic materials, reduces the crystallinity of cellulose, and partially degrades lignin [28]. Lignin not only shields cellulose microfibrils from the attack of hydrolytic enzymes but also binds and inactivates the latter [29]. The low lignin content in SBP, 3.82 % TS (Table 1), is advantageous and makes it a suitable substrate for biofuel production. However, a drawback of enzymatic hydrolysis is its long duration. Therefore, application of suitable enzyme preparations or their mixtures is necessary to quickly degrade the complex structure of lignocellulosic materials [30]. Our previous study [11] showed that the highest concentrations of reducing sugars were obtained when SBP was digested with a mixture of Celustar XL, which contains endo-glucanase and xylanase, and Agropect pomace, which contains pectinases (Table 1). According to Spagnuolo et al. [2], pectinolytic enzymes play very important role in degradation of SBP and other lignocellulosic materials because they improve an access of cellulases and hemicellulases to the substrates. Application of optimized combinations of hydrolytic enzymes enables to reduce their costs and achieve high yields of reducing sugars [30]. The extent of enzymatic hydrolysis may be increased by suitable pretreatment. It was reported that grinding of lignocellulosic biomass increased the yield of enzymatic hydrolysis by 5–25 % and reduced its time by 23–59 % [31]. The optimal size of particles after mechanical pretreatment depends on the type of biomass and its susceptibility to biodegradation. Also, the size of pores in lignocellulosic biomass, which decides of their penetration by enzymes, is one of main factors that influence the results of enzymatic hydrolysis [32].

Small particles (53–75 mm) of ground maize straw were 30 % more susceptible to enzymatic hydrolysis than the larger ones (425–710 mm), and the reduction of the size from 590 to 33 mm caused a 55 % increase in glucose concentration in the hydrolysates [33]. However, grinding of switch grass [34], pomace [35], and maize fibers [36] to 0.4 mm particles had a weak effect on the yield and rate of their enzymatic hydrolysis. The effect of SBP grinding on the yield of enzymatic hydrolysis has not been reported. The analysis of bar charts presented in Fig. 1 showed that grinding of SBP significantly increased the extent of its enzymatic conversion to reducing sugars. After the first day, their concentrations in the enzymatic hydrolysates of the ground (MEP) and not ground (EP) pulp reached 21.90 and 5.85 mg/mL, respectively. After 2 days of hydrolysis, the concentration of reducing sugars released from the ground SBP (MEP) increased to 36.20 mg/mL and was above fourfold higher than in the hydrolysate of not ground SBP (EP). After the next 2 days of hydrolysis, concentration of reducing sugars in the latter hydrolysate was increased by 44.0 % while in the hydrolysate of ground SBP (MEP), their concentration was virtually the same as after the first 2 days. Therefore, in further experiments enzymatic hydrolysis was conducted for 2 days (considered the optimum time).

Determination of sugar profiles of the SBP hydrolysates obtained by 2-day hydrolysis (Table 3) revealed that the hydrolysate of ground SBP (MEP) contained significantly more glucose (16.069 mg/mL) than the hydrolysate of not ground pulp (EP) (3.000 mg/mL). Both these hydrolysates contained also high concentrations of xylose (9.284 and 1.998 mg/mL for MEP and EP, respectively).

### Thermal-Pressure Pretreatment and Enzymatic Hydrolysis

**Fig. 2:**
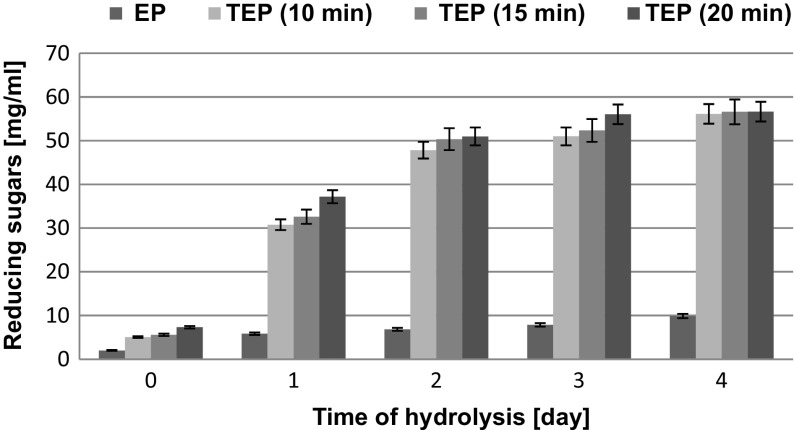
Changes in reducing sugars concentration during enzymatic hydrolysis of not ground SBP without and after thermal pretreatment

**Fig. 3:**
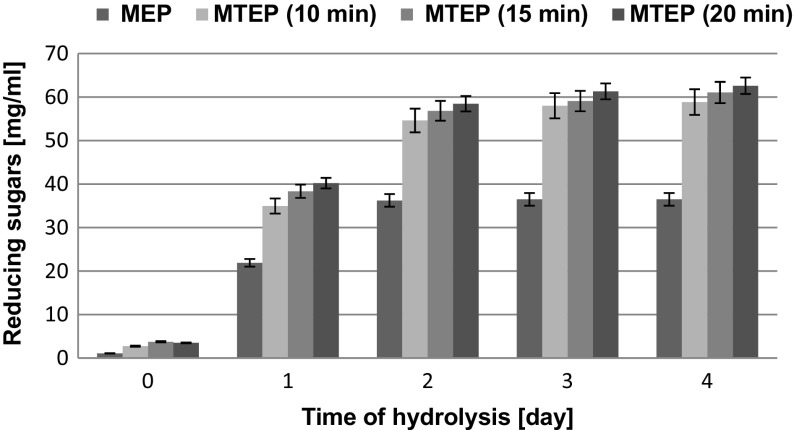
Changes in reducing sugars concentration during enzymatic hydrolysis of ground SBP without and after thermal pretreatment

The effect of thermal-pressure pretreatment of ground and not ground SBP on reducing sugars concentration in enzymatic hydrolysates is presented in Figs. 2 and 3 and in Table 3. The controls were enzymatic hydrolysates of either not pretreated (EP) or ground (MEP) pulp. The bar charts presented in Figs. 2 and 3 demonstrate that the thermal-pressure pretreatment positively affected the results of enzymatic conversion of the ground (MTEP) and not ground (TEP) pulp to reducing sugars. Even 10-min thermal-pressure pretreatment significantly increased the extent of enzymatic saccharification. However, when the time of processing was extended to 15 or 20 min, the ultimate concentration of reducing sugars in the enzymatic hydrolysates was not increased proportionally. Changes in reducing sugar concentrations in enzymatic hydrolysates of thermal-pressure pretreated not ground SBP (TEP) are presented in Fig. 2. Comparison of the presented data shows that when thermal-pressure pretreatment was conducted for 10 min, the concentration of reducing sugars in the enzymatic hydrolysate (TEP) was sevenfold higher than that in the control (EP). Irrespective of the time of thermal-pressure pretreatment of not ground pulp, the highest dynamics of enzymatic saccharification was observed within the first 2 days. After the second day of enzymatic hydrolysis, the concentration of reducing sugars in the hydrolysate of SBP subjected to 20-min thermal-pressure pretreatment was around 50.35 mg/mL while in the hydrolysates of SBP pretreated for 10 and 15 min, it was 5.0 and 2.8 % lower, respectively. After the next 2 days of enzymatic hydrolysis, the concentrations of reducing sugars in these hydrolysates were increased by 15.0 % on average.

Changes in concentrations of reducing sugars during hydrolysis of the ground and thermal-pressure pretreated SBP are shown in Fig. 3. Also in this case, saccharification dynamics was the highest within the first 2 days. When thermal-pressure pretreatment of ground SBP was conducted for 20 min, reducing sugars concentration reached 58.46 mg/mL within 2 days while in case of SBP processed for 15 and 10 min, it was 2.8 and 6.6 % lower, respectively. In comparison to the hydrolysate of pulp, which was not subjected to thermal-pressure pretreatment before enzymatic hydrolysis (MEP), these concentrations were 50.8, 56.8, and 61.4 % higher, respectively. After the next 2 days of enzymatic hydrolysis, the concentrations of reducing sugars increased by 10.0 % on average. On the basis of these results, it was decided that the optimum time of thermal-pressure pretreatment was 15 min.

The analysis of results presented in Table 3 showed that enzymatic hydrolysis of both ground and not ground SBP, which was subjected to 15 min thermal-pressure pretreatment, resulted in a mixture of simple sugars, containing primarily glucose (23–24 % *w*/*w*) and arabinose (11–12 % *w*/*w*) that were released from cellulose, hemicelluloses, and pectin, as well as smaller amounts of galactose, xylose, and mannose. After the first 2 days, concentrations of glucose were 24.765 and 23.116 mg/mL in the enzymatic hydrolysates of thermal-pressure pretreated ground and not ground SBP, respectively. Thus, grinding increased the yield of reducing sugars by only 10.4 % (Figs. 2 and 3) and because of its costs, it should be avoided before thermal-pressure pretreatment. However, grinding significantly increased the yield of reducing sugars in case of SBP which was subjected to enzymatic degradation without thermal-pressure pretreatment.

### Batch Anaerobic Digestion

**Fig. 4:**
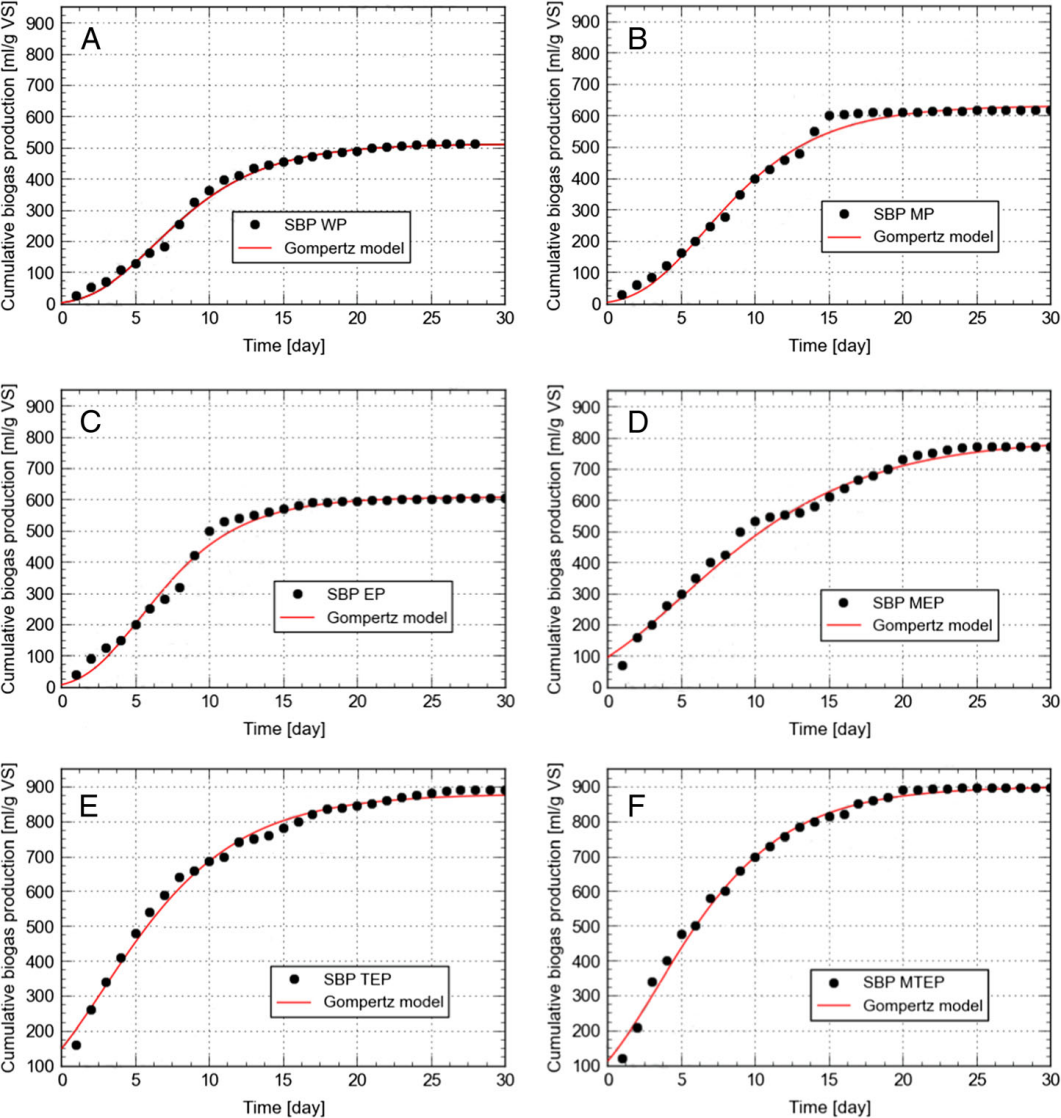
Cumulative biogas yields from batch anaerobic digestion of sugar beet pulp without and after pretreatment. Comparison of experimental results and data obtained using the Gompertz equation

The SBP samples, which were pretreated as described above, were subjected to anaerobic digestion to determine the influence of SBP pretreatment conditions on the dynamics of this process and biogas yield. SBP which was not pretreated before anaerobic digestion was the control (WP). The changes in parameters of batch anaerobic digestion are presented in Table 4 while the yields of biogas are shown in Fig. 4. These data demonstrate that both the course of anaerobic digestion and biogas yield depended on pretreatment conditions because the latter decided of the extent of pulp degradation to reducing sugars (Table 3). SBP is one of lignocellulosic wastes [2], which are recalcitrant to biodegradation under anaerobic conditions [6]. Therefore, it is often pretreated before anaerobic digestion [26]. The results presented in Table 4 and Fig. 4 demonstrate that grinding of SBP to around 2.5 mm particles (MP) resulted in 20.2 % increase in the yield of biogas compared to the control (WP).

**Table 4:** Parameters of anaerobic digestion

	TS (%)	VS (% TS)	Biogas yield (mL/g VS)	Methane yield (mL/g VS)	CH_4_/CO_2_	VS removal (%)	pH initial	pH final
WP	10.2	93.3	513.5 D (1.6)	277.3 D (2.4)	1.25	52.0	7.18	6.05
MP	10.0	93.4	617.2 C (6.3)	340.7 C (4.4)	1.33	58.2	7.20	6.17
EP	9.98	94.5	605.9 C (4.0)	351.4 C (3.7)	1.47	60.0	7.19	6.20
MEP	9.87	94.6	771.5 B (6.3)	452.1 B (4.3)	1.51	66.2	7.20	6.95
TEP	9.82	95.4	890.5 A (9.2)	534.3 A (3.9)	1.62	76.8	7.18	6.90
MTEP	9.68	95.9	898.7 A (15.7)	543.7 A (8.8)	1.66	78.0	7.19	6.92

Different letters (A, B, C, D) within the same parameters indicate statistical differences (*p* < 0.05). The values in brackets mean standard deviation

*WP* without pretreatment (SBP not ground), *MP* after mechanical pretreatment (SBP ground), *EP* after enzymatic pretreatment, *MEP* after mechanical and enzymatic pretreatment, *TEP* after thermal and enzymatic pretreatment, *MTEP* after mechanical, thermal, and enzymatic pretreatment

Comparison of the characteristics of residues after anaerobic digestion showed that the decrease in VS was 11.9 % higher in the case of the ground SBP (MP) than that in the case of the control, which was not pretreated (WP). Despite the initial pH adjustment to around 7.0, this parameter was considerably reduced at the end of anaerobic digestion, to 6.17 and 6.05 for the samples MP and WP, respectively. According to literature, the optimum initial pH of substrates subjected to anaerobic digestion is 6.7–7.5 [37]. In the initial phase of this process it drops even below 6.0 because of accumulation of volatile fatty acids and carbon dioxide. When these intermediates are converted to a mixture of methane and carbon dioxide, pH is increased and maintained at a constant level. However, when the activity of microorganisms which conduct processes of hydrolysis and acetic acid production is too high, and the function of microorganisms responsible for methane synthesis is insufficient, pH is decreased that brings about termination of methanogenesis [38]. In addition, the decrease in pH may be caused by the specificity of batch processes because the depletion of carbon sources brings about disadvantageous changes in the C/N and C/P ratios that in turn reduce metabolic activity of methane-producing microorganisms. This leads to the rise in organic acids concentrations and a decrease in pH in a digester.

The data presented in Fig. 4a, b demonstrate that within the first 15 days, the mean rate of biogas production from the ground pulp (MP) was 40 L/kg s.m.o day and nearly 97.2 % of the cumulative biogas yield was produced (617.2 mL/g VS) (for comparison, from the not ground pulp (WP) only 87.6 %). These results mean that grinding of SBP increased the dynamics of its anaerobic digestion, and the activity of biogas-producing microorganisms was very high within the first 15 days of anaerobic digestion. Furthermore, grinding of SBP (MP) caused that the CH_4_/CO_2_ ratio was increased by 6.4 %, compared to not ground SBP (WP). These results are consistent with the data reported by Delgenes et al. [39] who found that grinding of solid wastes increased biogas yield by 5–25 %. Moreover, when the size of particles of bagasse and coconut fibers was reduced from 5 mm to less than 0.85 mm, the yield of biogas was increased by 30 % while grinding of hemp biomass caused a 15 % rise in biogas yield [40]. Also, Mshandete et al. [41] reported that mechanical comminution of plant fibers increased biogas yield by up to 20 %.

The results of this study show that also enzymatic hydrolysis of SBP affected the course of anaerobic digestion and increased biogas yield. Interestingly, the yield of biogas from the ground SBP (MP) was by 1.9 % higher than that from not ground SBP which was subjected to 2-day enzymatic hydrolysis (EP). Thus, optimization of the size of ground SBP particles enabled to achieve the similar biogas yield as enzymatic hydrolysis of SBP. When SBP was both ground and degraded by enzymes (MEP), the yield of biogas was significantly increased in comparison to the untreated pulp (WP, 50.2 %) and not ground, enzymatically degraded (EP) pulp (27.3 %).

The analysis of curves presented in Fig. 4 and data collected in Table 4 shows that the rate of biogas production was 38.0 mL/g VS and methane concentration was 58.0 % *v*/*v* within the first 15 days of anaerobic digestion of enzymatically degraded SBP (EP). Within this period of time, the yield of biogas reached 94.1 % of the cumulative biogas yield. For comparison, the yield of biogas within the first 15 days of anaerobic digestion of the ground and enzymatically degraded pulp (MEP) was 81.0 % of the cumulative biogas yield (the rate of production was 41.66 mL/g VS, and the CH_4_/CO_2_ ratio was 2.7 % higher compared to EP).

The results obtained in this study are consistent with literature data. For instance, Schimpf and Valbuena [42] reported that enzymatic digestion of plant biomass, which caused degradation of structural polysaccharides, resulted in up to 20 % greater biogas yield. Mshandete et al. [43] studied the effect of enzymatic pretreatment of *Agave sisalana* residues on the yield of batch anaerobic digestion and found that degradation of these wastes with hydrolases, such as cellulases, amylases, and xylanases, increased biogas yields by 26 % in comparison to the reference, which was not subjected to enzymatic hydrolysis before anaerobic digestion. Conversely, Romano et al. [44] who subjected wheat straw (Jose Tall variety) to enzymatic degradation before anaerobic digestion did not observe any increase in the yield of biogas and methane, in comparison to the digestion of not pretreated straw.

In the next step, biogas was produced from SBP which was subjected to thermal-pressure pretreatment and enzymatic hydrolysis (TEP) or ground, thermal-pressure pretreated and digested by the enzymes (MTEP). The maximum cumulative biogas yield, 898.7 mL/g VS, was derived from the ground, thermal-pressure pretreated and enzymatically hydrolyzed SBP (MTEP). This yield was 75.0 % higher compared to the yield obtained from the control (WP). When SBP was thermal-pressure pretreated and digested by enzymes (TEP), the yield of biogas was only slightly lower (890.5 mL/g VS) than the maximum yield (0.9 %), and was 73.4 % higher compared to the control (WP). The analysis of the residues after anaerobic digestion showed that the highest decrease in VS (78.0 %) was observed in case of the sample MTEP while in case of the sample TEP, it was 1.5 % smaller. The ultimate pH of these residues was 6.92 and 6.90 for MTEP and TEP, respectively (slightly lower than in case of MEP, 6.95). The results obtained prove that the simple sugars released by the thermal-pressure hydrolysis from SBP had a positive impact on the activity of methane-producing bacteria within 30 days of anaerobic digestion.

The data presented in Fig. 4 and Table 4 demonstrate that biogas obtained from the sample MTEP was characterized by the highest CH_4_/CO_2_ ratio (1.66), which was 2.4 and 9.0 % higher in comparison to biogas obtained from the samples ETP and MEP, respectively. Within the first 15 days of anaerobic digestion, the rate of biogas production from the samples MTEP and ETP was 54.66 and 52 mL/g VS (91.2 and 87.6 % of the cumulative biogas yields were obtained), respectively.

### Kinetics of Anaerobic Digestion

Kinetics of biogas production from SBP by batch anaerobic digestion was modeled using a modified Gompertz equation. The values of kinetic constants—*A*, *λ*, and *μ* (Table 5)—were determined by nonlinear regression. The simulation of expected biogas yield, using selected experimental data and Gompertz equation, is presented in Fig. 4. The analysis of data, which are shown in Table 5, leads to a conclusion that the values of kinetic constants depended on SBP pretreatment conditions. Pretreatment of SBP caused an increase in the potential biogas yield (*A*) and maximum rate of biogas formation (*μ*) while the lag phase (*λ*, the time between inoculation and biogas appearance) was reduced. The longest lag phase *λ*, 2.038 days, was observed when SBP was not pretreated (WP). This value was correlated with the high content of polysaccharides, accounting for around 83.6 % SBP dry weight (Table 1). According to Krishania et al. [45], the complex three-dimensional structure of lignocelluloses retards biogas production by anaerobic microorganisms in the first days of anaerobic digestion. Grinding of SBP (MP) caused a decrease in the value of λ, to 1.533 day while enzymatic hydrolysis reduced the lag phase by 49.5 and 95.9 % for not ground (EP) and ground (MEP) pulp, respectively. Thermal-pressure pretreatment and enzymatic hydrolysis of ground (MTEP) and not ground (TEP) pulp reduced the lag phase to 0 days that means that biogas production begun almost immediately after inoculation. The positive impact of thermal pretreatment on biogas production was also reported by Cano et al. [46] who observed that thermal pretreatment of brewer’s spent grain increased biogas yield and reduced the lag phase from 0.8 to 0 days.

**Table 5:** Kinetic constants of biogas production

	*A* (mL/g VS)	*μ* (mL/g VS day)	*λ* (day)	*R* ^2^
WP	515.271	45.493	2.038	0.996
MP	639.523	52.935	1.533	0.995
EP	610.806	59.552	1.028	0.994
MEP	800.094	60.384	0.062	0.991
TEP	882.019	63.928	0	0.996
MTEP	903.289	69.451	0	0.996

*A* biogas production potential (mL/g VS), *μ* maximum biogas production rate (mL/g VS day), *λ* minimum time to produce biogas (days), *R* ^2^ correlation coefficient

The modified Gompertz model fitted well to the experimental results, which is shown in Fig. 4 since values of the correlation coefficient *R* ^2^ varied from 0.991 to 0.996. According to this model, the highest potential biogas yield, *A*, 903.289 mL/g VS, could be obtained from the ground, thermal-pressure pretreated and enzyme degraded (MTEP) pulp. This value was correlated with the maximum biogas production rate (*μ*) of 69.451 mL/g VS day. These values of *A* and *μ* were 11.4 and 13.1 % higher than those obtained for the ground and enzymatically degraded (MEP) pulp. Thus, also according to the model, the yield of biogas after the three step pretreatment (MTEP) of pulp was only slightly higher compared to that after the two-step pretreatment (MEP). Like in case of the experimental results, the expected yield of biogas from the ground pulp (MP), 639.523 mL/g VS, was 4.7 % higher than from the enzyme treated pulp (EP) while the maximum rate of biogas production was 11.1 % higher for EP (59.552 mL/gVS day) than for MP. Thus, optimization of the size of ground SBP may give the similar effect as enzymatic hydrolysis of this material. However, the presence of active hydrolytic enzymes in the hydrolysate may positively affect the rate of biogas production.

The lowest values of *A* and *μ* (515.271 and 45.493 mL/g VS day, respectively) were obtained for SBP, which was not pretreated before anaerobic digestion (WP). These results provide additional evidence that SBP requires pretreatment before anaerobic digestion.

### Process Energy Requirements and Gain Power

**Fig. 5:**
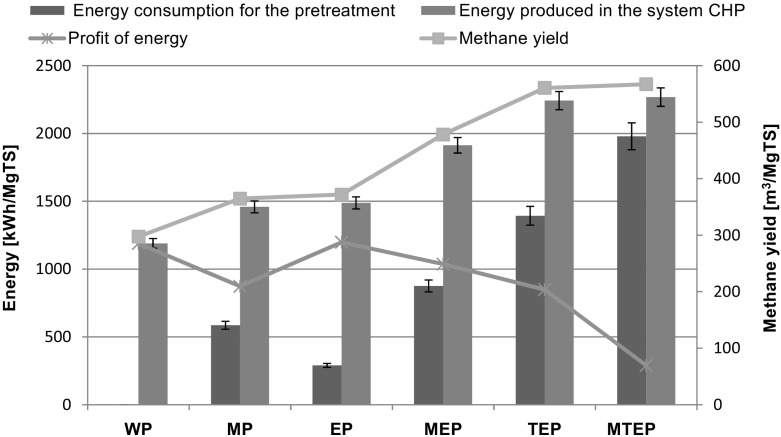
The relationship between the energy consumed for SBP pretreatment and of the electric energy gain from incineration of biogas in CHP

The relationship between the energy consumed for SBP pretreatment (kWh/Mg TS) under laboratory conditions and the expected value of the extra net electric energy gain from incineration of biogas in a cogeneration system is presented in Fig. 5. Selling the electric energy is the principal source of income for the majority of agricultural biogas plants, and therefore, only this kind of energy was considered in the economic analysis while the heat produced by biogas incineration was not taken into consideration. The bar charts presented in Fig. 5 demonstrate that conditions of SBP pretreatment had a strong impact on biogas yield. The increase in the number of technological operations significantly reduced the extra net gain of electric energy, being the difference between the electric energy produced in the cogeneration system and the energy requirements for pretreatment. The highest methane yields from 1 kg TS were obtained from the samples TEP and MTEP (560.53 and 567.13 m^3^/MgTS, respectively). Incineration of these amounts of methane in the CHP system enabled to produce 2242.12 and 2268.52 kWh/Mg TS electric energy, respectively. However, because of the greater energy requirement values for pretreatment in case of the samples TEP and MTEP, compared to the control WP, the extra net electric energy gains were 24.4 and 75.6 % lower, respectively. SBP pretreatment by either grinding or enzymatic hydrolysis caused that biogas yields from the samples MP and EP were 22.7 and 25.0 % higher than from the control WP, respectively. The energy input for grinding, 585.93 kWh/Mg TS, was 50.5 % higher than that for enzymatic hydrolysis, while the electric energy gains from the samples EP and MP were similar, 1487.52 and 1459.00 kWh/Mg TS, respectively. Because of the low energy input for enzymatic hydrolysis, the electric energy gain from the samples EP and WP was similar, 1189.56 and 1197.52 kWh/Mg TS, while for the sample MP, it was 26.6 % lower compared to that from WP. Interestingly, the values of the extra net electric energy gain from the samples MP and TEP were similar although the yield of methane from TEP was 53.7 % higher than from MP.

Comparison of the extra net electric energy gain values suggests that enzymatic hydrolysis is a suitable method of SBP pretreatment before anaerobic digestion. Also, grinding and its combination with enzymatic hydrolysis of SBP seem to be cost-effective. Grinding costs depend on the ultimate size of SBP particles and a type of mill while the costs of enzymatic hydrolysis are affected by the costs of enzymes and duration of this process. Reassuming, the optimal SBP pretreatment conditions before anaerobic digestion must compromise between the low energy requirements and the high yield of biogas for electricity production. Furthermore, the whole process has to be economically feasible in industrial scale.

## Conclusion

Methods such as grinding, thermal-pressure pretreatment, and enzymatic hydrolysis, as well as their combinations, significantly increased biogas yields from anaerobic digestion of SBP. The highest cumulative biogas productivity, 898.7 mL/gVS, was obtained from enzymatic hydrolysates of the ground and thermal-pressure pretreated SBP. This value was slightly higher compared to the biogas yield from enzymatic hydrolysates of thermal-pressure pretreated but not ground SBP (890.5 mL/g VS). The yield of biogas from the ground SBP (617.2 mL/g VS) was similar to that from the 2-day enzymatic hydrolysate of not ground SBP. The modified Gompertz model fitted well to the experimental results obtained in this study.
